# Stockperson attitudes towards Maternity Rings and farrowing crates

**DOI:** 10.3389/fvets.2025.1579263

**Published:** 2025-07-23

**Authors:** Lauren M. Staveley, Kate J. Plush, David S. Lines, Lauren M. Hemsworth, Darryl N. D’Souza, Robert J. van Barneveld

**Affiliations:** ^1^SunPork Group, Eagle Farm, QLD, Australia; ^2^Animal Welfare Science Centre, Faculty of Science, The University of Melbourne, Parkville, VIC, Australia

**Keywords:** stockperson, maternity ring, attitude, welfare, farrowing

## Abstract

Societal attitudes suggest low support for confinement housing in livestock farming, such as the farrowing crate. The attitudes of stockpersons working in these systems are yet to be understood but should be prioritised as their human-animal interactions have significant effects on animal welfare. The aim of this investigation was to explore the attitudes of stockpeople employed on pig farms with experience working in both free-farrowing and farrowing crate systems, and to better understand the contributing factors that shape these attitudes. An anonymous survey was conducted across four pig breeder farms with both Maternity Rings (MR) and farrowing crates (FC) installed. A total of 86 stockpeople volunteered to participate. The survey consisted of an opinion-based rating of sow welfare that considered four specific behaviours, and two attitude-based questionnaires. The composite score of sow welfare was higher in a MR when compared to a FC (39.8 ± 0.87 versus 28.0 ± 0.87, *p* < 0.05), regardless of attitude towards working with sows in different lactation housing systems. Stockpeople that believed FC systems would always be necessary were more likely to avoid interactions with difficult pigs (r(_84_) = 0.327, *p* = 0.005), and more likely to rate piglet welfare as more important than sow welfare (r(_84_) = 0.380, *p* = 0.001). In contrast, stockpeople that were confident in their abilities and understandings of sow behaviour were more likely to rate the sows welfare higher in a MR (r(_84_) = 0.339, *p* = 0.002) and believed that it provided an environment that enabled the sow to better interact with her piglets (r(_77_) = 0.434, *p* < 0.001). Stockpersons that were more likely to interact with pigs (r(_84_) = 0.322, *p* = 0.011) and were more satisfied with their job (*β* = 0.341, *p* = 0.003) were more likely to rate sow welfare higher in a MR. Overall, stockpeople rated sow welfare higher in a MR in comparison to a FC. The main driver of negative attitudes towards a MR appeared to be a lack of understanding of sow behaviour. If we can develop ways to modify stockperson behaviour to improve sow and piglet welfare outcomes, we have a better chance of introducing alternative farrowing systems.

## 1 Introduction

Farrowing crates were introduced in the 1960’s (1) with the intention of reducing live-born piglet mortality as they allowed for control over sow postural changes, while providing greater efficiency and safety to stockpeople. While the farrowing crate has provided benefits for piglet survival, it does impose physical restrictions impacting sow welfare. There are two periods when the confinement of the sow within a farrowing crate has high welfare constraints (i) prior to farrowing when the sow has an intrinsic need to build a nest and (ii) as lactation proceeds when the sow begins to wean her litter.

A significant body of research has been directed towards alternatives to the farrowing crate that address the welfare issues sows encounter, as well as the higher live-born piglet mortality in free-farrowing systems (2–4). This research has looked at various design aspects affecting sow and piglet behaviour in these free-farrowing systems to improve the live-pig mortality. Whilst the design of free-farrowing pens is a key factor impacting production performance, the role of the stockperson is also critical in how the sow and piglets are managed, and this has received little attention.

Several studies have quantified the views of the general public and have found that confinement housing such as the farrowing crate have low societal support (5), with consumer preference for provision of more ‘natural’ living conditions (6–8). In contrast people with links to agriculture are more likely to have a positive attitude towards a farrowing crate (5). However, the attitudes of the stockperson working daily with animals housed in these farrowing systems, whether they be farrowing crates or free-farrowing systems, requires further investigation.

Interactions between intensively farmed animals and the stockpeople responsible for their care have significant effects on animal health, welfare and productivity (9–12). The quality of these human-animal interactions is dependent on the stockperson’s behaviour towards the animal (13, 14). According to the Theory of Planned Behaviour (15), understanding a particular attitude can lead to a path of change in human behaviour. While attitudes are dependent on an individual’s behavioural, normative and control beliefs, there are numerous studies which describe the positive impact that training can have on changing attitudes and the subsequent behaviour of stockpeople (9, 16–20). It is this attitudinal shift that improves interactions between stockpeople and animals, increasing welfare and production (11, 21–26). Given this, the attitudes of stockpeople that work with pigs in farrowing and lactation in free-farrowing systems should be better understood.

The Maternity Ring (MR) system was developed as a free-farrowing alternative to conventional farrowing crates that, unlike other commercially available options, preserved space similar to that of a crate, while providing the sow the ability to turn around before, during and after farrowing. Plush et al. (27) has demonstrated that the MR farrowing system was able to improve the welfare of the sow during farrowing and lactation. The aim of this study was to quantify the attitudes of stockpeople employed on pig farms with experience working with the MR and farrowing crate systems, and to better understand the contributing factors that shape these attitudes.

## 2 Materials and methods

**Figure 1:**
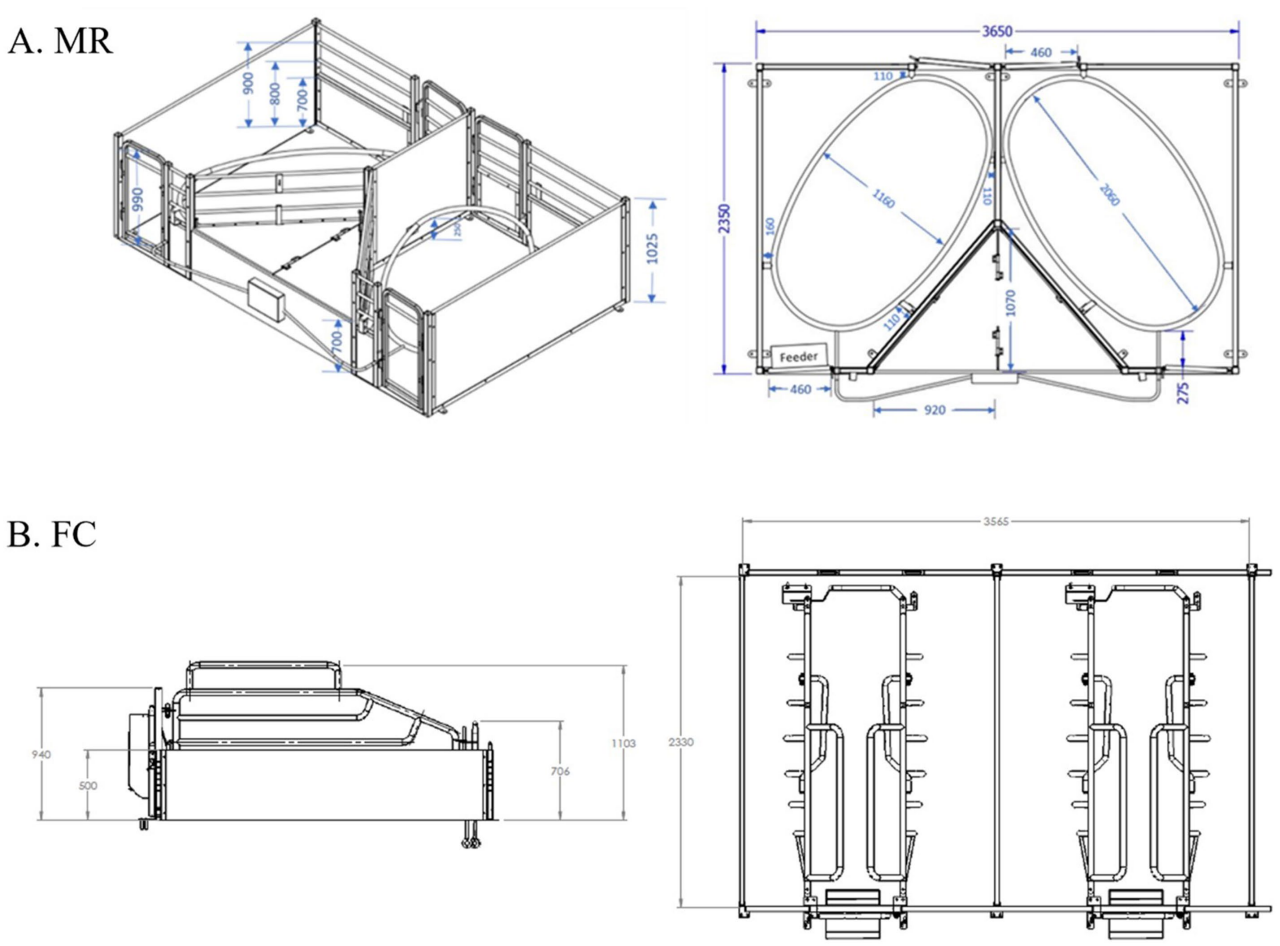
Dimensions of key design features of the **(A)** Maternity Ring (MR) and **(B)** farrowing crate [FC; (27)].

Ethics clearance for research with human subjects was obtained via The University of Melbourne’s Human Ethics Advisory Group (Ethics ID: 2022–25,237–35,354-4). The survey was conducted at four South Australian farms that had both Maternity Rings (MR) and farrowing crates (FC) with farm 1 *n* = 160 MR and 30 FC, farm 2 *n* = 80 MR and 620 FC, farm 3 *n* = 160 MR and 680 FC and farm 4 *n* = 120 MR and 1,480 FC. The MR was a close-confinement free system 1,800 mm in width by 2,350 mm in length with a ring installed on the diagonal with dimensions 1,160 mm in width and 2,060 in length and height of 250 mm from flooring [Figure 1; (27)]. The FC was 1,783 mm in width by 2,330 mm in length with a crate installed parallel to the external dividers measuring 720 mm in width and 2,330 mm in length which restricted the sows movement.

Prior to the presentation of the survey, participants were given a plain language statement (i.e., an explanatory statement outlining the research aims), advised that participation was entirely voluntary and that they could withdraw at any time if so desired, and then written consent was sought and obtained. A total of 86 from the 110 stockpeople working across these farms volunteered to participate in this study. The survey was delivered in a classroom presentation style, where the lead researcher displayed each question and its associated Likert scale on a projector, while reading each question aloud. This allowed participants to clarify anything they were uncertain of and for the researcher to follow up any questions for clarification. Although the questionnaire was delivered in groups, participants completed the questionnaire individually with no group discussion of answers taking place. The completion of the survey took between 30 to 60 min, depending on the number of clarifying questions asked. The survey was made up of a opinion-based assessment of sow welfare and two questionnaires.

### 2.1 Opinion-based assessment of sow welfare

Welfare was scored by playing the participants eight short videos of a sow performing the four behaviours listed in Table 1 in both a FC and MR. Stockpeople were asked to use their experience and understanding of pig behaviour to respond to a series of questions to define the perceived welfare state of the sow using the video as a reference. Videos from both FC and MR sows were randomly selected from a pre-existing catalogue that contained footage collected 18 h prior to farrowing and 4 h on day 5 of lactation and scanned to the first time the listed behaviour (Table 1) was observed. Using a five-point Likert scale, stockpeople were asked to indicate their level of agreement with the list of terms (e.g., score 1 = strongly agree with one or more poor welfare descriptor, 5 = strongly agree with one or more high welfare descriptor listed in Table 1). The scores for each behaviour were then summed to give a composite sow welfare assessment, with a maximum score of 50 representing the highest welfare outcome.

**Table 1:** Opinion-based assessment of sow welfare as presented to stockpeople.

Behaviour	Question	Poor welfare descriptor	High welfare descriptor
Nesting	How would you classify the sows demeanour?	Agitated, tense, annoyed, frustrated, aggravated, stressed	Alert, playful, satisfied
	How well do you think the sow is able to express her natural behaviour?	Not very well	Very well
Lying down nursing	How would you rate the welfare of this sow?	Low	High
	How easily is this sow able to perform the desired behaviour?	Difficult	Easy
	How do you think this sow feels?	Agitated, annoyed, frustrated, impatient, irritated	Alert, calm, relaxed, content, comfortable, satisfied
Postural change	How would you rate the welfare of this sow?	Low	High
	How easily is this sow able to perform the desired behaviour?	Difficult	Easy
	How do you think this sow feels?	Agitated, annoyed, frustrated, impatient, irritated	Alert, calm, relaxed, content, comfortable, satisfied
Interaction with piglets	How do you think this interaction makes the sow feel?	Annoyed, frustrated, impatient, disruptive, grumpy	Calm, relaxed, content, maternal, satisfied
	How well is the sow able to interact with her piglets?	Difficult	Easy

### 2.2 Questionnaires

Two questionnaires were used to accurately capture stockperson attitudes and opinions towards working with sows in MR and FC systems (included in Supplementary material). The first questionnaire focused on stockperson attitudes, and the second aimed to capture attitudes towards working with pigs in different housing systems. The questionnaires were adapted from previous work conducted in livestock [see (28–31)]. Using a five-point Likert scale, stockpeople were asked to indicate their level of agreement to the statements, the level of importance or the perceived difficulty of performing an activity (e.g., score 1 = disagree/not at all important/not difficult to score, 5 = strongly agree/very important/very difficult). Questions were framed as both positive and negative, with responses recoded so that a high score indicated that participant held a positive attitude towards working with pigs. Several statements on a specific topic were used to measure consistent beliefs, which allowed the identification of a person’s attitude towards the specific topic (32).

The first attitude questionnaire involved a total of 47 questions and was adapted to target attitudes towards working with pigs, husbandry practices, and assessing the participants overall confidence and knowledge of pig behaviour. The sections of the questionnaire are reported in Table 2.

**Table 2:** Structure of attitude-based questionnaire.

Section	Information gathered
Demographics	Gender, farm identification, experience in the pig industry, experience with different farm sections and preferences
Attitude towards pigs	General perceptions of pigs at different cycle stages, general attitudes towards working with pigs, normative and behavioral beliefs in relation to daily husbandry
Attitude towards job	General attitudes towards their role, normative and behavioral beliefs in relation to their role
Confidence in own knowledge and abilities	Control beliefs in relation to their influence and role

The second questionnaire was opinion-based, consisting of 29 questions around the participants’ attitudes to working with sows in MR or FC systems. Again, several statements on a specific topic were used to measure consistent beliefs, which allowed the identification of a person’s attitude towards a specific topic (32).

### 2.3 Statistical analysis

All data were analysed in SPSS (v28 IBM, Armonk, NY, USA) with *p* < 0.05 achieving significance and *p* < 0.10 a trend. The impact of farrowing accommodation type on composite opinion-based sow welfare assessment was analysed using linear regression, with the contribution of each of the four individual behaviours (nesting, lying, changing posture and interacting with piglets) using ordinal regression. A Principal Component Analysis (PCA) was carried out, using a correlation matrix on the composite opinion-based sow welfare score, with two components that related to farrowing accommodation type created. Survey data were analysed using PCA, followed by an Oblimin rotation, to identify commonalities amongst the survey items, with Cronbach’s alpha presented as a measure of internal consistency demonstrating how closely related the questions were as a group. Attitude components were assigned labels that reflected the attitude items which formed the components. The suitability of the data for the analyses was assessed using criteria outlined by Pallant (33); the correlation matrix coefficients were all above the required 0.3, the Kaiser-Meyer-Olkin (KMO) values exceeded the recommended value of 0.6, and Bartlett’s Test of Sphericity reached statistical significance. Questions that were established as belonging to a common underlying component were then summed to produce a composite score for that component. Scale reliabilities were measured using Cronbach’s *α* coefficients with an α > = 0.70 as the criterion for acceptable reliability (34). Questions were included in a scale if their loading on the relevant component exceeded 0.33 (35) and if, on the basis of face validity, they could be summarized by just one construct. Correlations between composite variables identified from PCA on the demographics, attitudes towards pigs, attitudes towards job, confidence in own knowledge and abilities, and their opinions of MR vs. FC was conducted using Pearsons product moment correlations. Separate stepwise multiple linear regressions were used to identify those demographic variables that predicted each of the behaviours of interest reported in the first questionnaire (see Table 3).

**Table 3:** Components from the questionnaires grouped into composite scores, a high score indicating a strong agreement to the statements.

Assigned attitude component label	Cronbach’s alpha	Questions
Positive opinion of pigs	0.77	Pigs are easy to work with Pigs are friendly to people Pigs are intelligent
Negative opinion of pigs	0.65	Pigs are gluttons Pigs are dirty
Likelihood of pig interactions	0.81	I frequently talk to or pat pigs that are in-oestrous I frequently talk to or pat pigs that are not in-oestrous I frequently pat and talk to sows in the farrowing house that are calm/happy I frequently pat and talk to sows in the farrowing house that are protective
Avoidance of difficult pigs	0.59	I find talking to protective sows in the farrowing house does not help with daily tasks I avoid protective sows in the farrowing house I find talking to in-oestrous pigs does not help with daily tasks I find in-oestrous pigs frustrating
Effort required working with difficult sows	0.83	Verbal/physical effort/difficulty is required to move pigs in-oestrous Verbal/physical effort/difficulty is required perform routine husbandry in farrowing around protective sows
Effort required working with calm sows	0.88	Verbal/physical effort/difficulty is required to move pigs not in-oestrous Verbal/physical effort/difficulty is required perform routine husbandry in farrowing around calm/friendly sows
Job satisfaction	0.39	How interesting is your job? Would you attend training courses in your own time if they were available?
Excitement about breaks	0.14	I look forward to the end of the working day I look forward to smoko/lunch breaks?
Likelihood of retention in industry	0.57	Do you think you’ll be in the pig industry in 5 years? Do you think you’ll be in the pig industry in 10 years? How often do you discuss work methods during smoko/lunch breaks?
Self confidence in knowledge	0.77	I know a lot about factors which affect reproduction in pigs I know a lot about diseases in pigs I understand pig behaviour well
FC defender	0.75	I believe farrowing crates will always be necessary I would be proud to show my friends/family around a farrowing house with only farrowing crates I believe farrowing crates are better for the sow’s welfare I believe farrowing crates are better for the piglet’s welfare
FC difficulty	0.65	How difficult is weaning in a crate? How difficult is assisting a farrowing sow in a crate?
FC preference	0.67	I prefer working with sows in farrowing crates I feel safe working in farrowing crates I believe detecting sows that need medical intervention is easier in a farrowing crate
MR defender/preference	0.80	I believe free-farrowing is the future of the industry I prefer weaning in Maternity Rings I prefer working with sows in Maternity Rings I feel safe working in Maternity Rings I believe detecting sows that need medical intervention is easier in a Maternity Ring I would be proud to show my friends/family around a farrowing house with only Maternity Rings I believe Maternity Rings are better for the piglet’s welfare
MR difficulty	0.68	How difficult is weaning in a Maternity Ring? How difficult is assisting a farrowing sow in a Maternity Ring?
Confidence working in a MR	0.50	I believe I understand sow behaviour well enough to work safely in a Maternity Ring I believe free-farrowing is better for the sow’s welfare
		I believe the sow welfare is more important than piglet welfare
		I believe piglet welfare is more important than sow welfare

The opinion-based sow welfare scoring of sows in MR or FC was subject to the same analyses as the previous questionnaires with components presented in Table 4.

**Table 4:** Components from the opinion-based sow welfare assessment grouped into composite scores, a high score indicating a more positive response to the question.

Assigned attitude component label	Cronbach’s alpha	Associated video	Questions
MR sow welfare	0.82	Sow in a MR lying down nursing, posture change from standing to lying and nesting with hessian prior to farrowing	How would you rate the welfare of this sow? How do you think this sow feels? How easily is this sow able to perform the desired behaviour? How well do you think the sow is able to express her natural behaviour? How would you classify the sow’s demeanour?
FC sow welfare	0.87	Sow in a FC lying down nursing, posture change from standing to lying and nesting with hessian prior to farrowing	
MR, ability to interact with piglets	0.81	Sow in a MR interaction with piglets during farrowing	How do you think this interaction makes the sow feel? How well is the sow able to interact with her piglets?
FC, ability to interact with piglets	0.92	Sow in a FC interaction with piglets during farrowing	

## 3 Results

**Figure 2:**
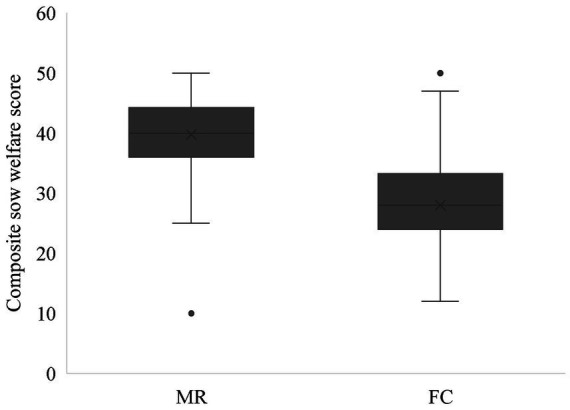
Mean (x) ± SEM opinion-based assessment of sow welfare using four specific behaviours (nesting, lying, changing posture and interacting with piglets) when housed in either a MR or a FC *p* < 0.001.

Participants average length of experience working with pigs was 9.0 ± 1.04 years, 62.8% of the participants were male (*n* = 54), 33.7% female (*n* = 29) and 3.5% of the participants declined to answer (*n* = 3). Overall, stockperson opinion of sow welfare was 39.8 ± 0.87 in MR and 28.0 ± 0.87 in FC (Figure 2; *p* < 0.001). The sow being housed in a FC was associated with a lower welfare score for nesting (odds ratio 0.277 (95% CI 0.156–0.496), Wald *χ*^2^(1) = 18.747, *p* < 0.001), lying (odds ratio 0.258 (95% CI 0.143–0.157), Wald *χ*^2^(1) = 20.375, *p* < 0.001), changing posture (odds ratio 0.080 (95% CI 0.041–0.157), Wald *χ*^2^(1) = 53.414, *p* < 0.001) and interacting with piglets (odds ratio 0.110 (95% CI 0.055–0.205), Wald *χ*^2^(1) = 45.136, *p* < 0.001).

Relationships between opinion-based component scoring of sow welfare and stockpersons attitudes and opinions surveys are reported in Table 5 and were in the low to moderate range. The component label MR sow welfare was positively correlated with positive opinions of pigs, the likelihood of interactions with pigs, their job satisfaction, self confidence in their own knowledge, how confident they were and whether they preferred working in a MR. A negative relationship between this same label and likely to avoid interactions with difficult pigs and preferred working with crated sows was also established. The component label FC sow welfare was positively correlated with positive opinions of pigs, likely to avoid interactions with difficult pigs, FC would always be necessary and piglet welfare was more important than sow welfare.

**Table 5:** Pearsons’s correlation coefficients between opinion-based sow welfare components and stockpersons attitudes and opinions surveys*.

Assigned attitude component label	MR sow welfare	FC sow welfare	MR, ability to interact with piglets	FC, ability to interact with piglets
Positive opinion of pigs	**0.392**	**0.404**	0.125	0.073
Negative opinion of pigs	−0.105	0.049	−0.043	−0.181
Likelihood of pig interactions	**0.322**	−0.121	**0.269**	−0.022
Avoidance of difficult pigs	**−0.423**	**0.311**	**−0.403**	0.151
Effort required working with difficult sows	0.229	0.137	0.079	0.046
Effort required working with calm sows	−0.056	0.116	0.081	0.168
Job satisfaction	**0.226**	0.033	0.114	−0.036
Excitement about breaks	0.069	−0.216	−0.153	−0.067
Likelihood of retention in industry	−0.069	0.199	0.026	−0.008
Self confidence in knowledge	**0.261**	−0.072	**0.281**	−0.013
FC defender	−0.241	**0.497**	**−0.403**	**0.449**
FC preference	**−0.269**	0.031	0.031	−0.115
MR defender/preference	**0.331**	0.148	0.028	−0.125
FC difficulty	0.192	0.01	*−*0.054	0.08
MR difficulty	0.124	−0.006	−0.202	−0.082
Confidence working in MR	**0.615**	−0.235	**0.434**	**−0.291**
Sow welfare > piglet	0.223	−0.177	0.124	**−0.269**
Piglet welfare > sow	−0.049	**0.253**	−0.216	0.21

*Bold value denotes significance at *p* < 0.05.

The way a participant scored a sow’s ability to interact with her piglets in a MR and how they perceived this interaction made the sow feel, was positively correlated with the likelihood of that person interacting with pigs, their self confidence in their own knowledge and their confidence in working in a MR system. Participants that were more likely to avoid interactions with difficult pigs and believed FC would always be necessary, were more likely to give a lower score for a sows ability to interact with her piglets in a MR and believed that this interaction was not satisfying for the sow (Table 5). How a participant scored a sow’s ability to interact with her piglets in a FC and how this interaction made her feel, was positively correlated with their self confidence in their own knowledge. Participants that were more likely to score the welfare effect of this interaction as higher were also less confident in their own knowledge and less likely to rate the sow’s welfare as more important than the piglets welfare.

When all composite variables for both attitudes and opinions surveys were entered into a linear regression model with the component label MR sow welfare as the dependent variable, one variable was predictive of this preference (Confidence working in a MR) and accounted for 40.1% of its variance (Table 6). Stockpeople that believed they understood sow behaviour well enough to safely work in a MR had a higher MR sow welfare score. When all composite variables for the attitudes and opinions surveys were entered into a linear regression model with the component label FC sow welfare as the dependent variable, one variable was predictive of this preference (FC defender) and accounted for 41.4% of its variance. Stockpeople that believed farrowing crates will always be necessary had a higher FC sow welfare score.

**Table 6:** Linear regression with the predictive factor of composite variables for stockpersons attitudes and opinions survey and dependent variable of opinion-based sow welfare score.

Predictive factor	Dependent variable	R^2^	Beta coefficient (standardised)	t	*p-value*
Confidence working in a MR	MR sow welfare	0.401	0.634	5.733	< 0.001
FC defender	FC sow welfare	0.414	0.643	5.882	< 0.001
Confidence working in a MR	Ability to interact with piglets - MR	0.313	0.285	2.341	0.023
FC defender		0.313	−0.318	−2.665	0.010
Likelihood of pig interactions		0.313	0.233	2.008	0.050
FC defender	Ability to interact with piglets - FC	0.230	0.480	4.093	< 0.001

When all composite variables for both attitudes and opinions survey were entered into a linear regression model with the component label ‘Ability to interact with piglets – MR’ as the dependent variable, three variables were predictive of this preference (Confidence working in MR, Likelihood of pig interactions and negatively with FC defender) and accounted for 31.3% of its variance. Stockpeople that believed they understood sow behaviour well enough to work safely in a MR and that were more likely to interact with pigs in general rated the sow’s ability to interact with her piglets and the positive effect this had on the sow’s welfare as higher. In comparison, stockpeople that believed that farrowing crates would always be necessary were more likely to rate the sow’s ability to interact with her piglets in a MR as lower and that this had a diminished effect on her welfare. When all composite variables for both attitudes and opinions survey were entered into a linear regression model with the component label Ability to interact with piglets – FC as the dependent variable, one variable was predictive of this preference (FC defender) and accounted for 23.0% of its variance. Stockpeople that believed FC would always be necessary were more likely to rate the sow’s ability to interact with her piglets in a FC as higher, with this having a positive effect on her welfare.

When all composite variables for both questionnaires were entered into a linear regression model with ‘MR defender/preference’ as the dependent variable, one variable was predictive of this preference (Job satisfaction) and accounted for 11.7% of its variance (R^2^ = 0.114, *t* = 2.86). Stockpeople that were more satisfied with their job were more supportive of the MR (*β* = 0.341, *p* = 0.003). When all composite variables for both questionnaires were entered into a linear regression model with ‘FC defender’ as the dependent variable, no variable was predictive of this attitude.

## 4 Discussion

Free-farrowing systems when compared to FC require a higher level of human interaction with the sow (36), therefore, the experiences of the stockperson working in these systems should be explored. The first step in investigating this is to better understand the attitudes and opinions of stockpeople that have experience working in both systems. Using opinion-based sow welfare assessment, stockpeople in the current study ranked the welfare of sows higher in a free-farrowing system (MR) when compared to a confined housing system (FC). The results suggest several key attitudes and beliefs influenced this assessment. Stockpeople that viewed sow welfare positively in a MR were confident in their knowledge of sow behaviour and felt this contributed to a safer working environment compared to those that viewed sow welfare positively in a FC and deemed crates as being indispensable for pig farming. A lack of confidence working in free-farrowing systems, stemming from a lack of understanding of sow behaviour appears to be a barrier to staff support for these systems. Given this finding, the development of a targeted training program may prove beneficial in supporting positive attitudes towards free-farrowing systems in the future.

In the current investigation, stockpeople were shown video footage of sows in a MR and FC and concentrated on four specific behaviours. These behaviours were selected from literature as being divergent in free-farrowing and farrowing crate systems and impacting welfare. The ability of the sow to nest and interact with piglets (27), and ease with which the sow can change posture and nurse her litter (37) are key behaviours which are inhibited or impaired in farrowing crate systems. The opinion-based assessment rated welfare higher when a sow was housed in a MR compared to a FC both individually and when all behaviours were compiled into a composite score. Recognising the importance of these behaviours to sow welfare may lead to more positive stockperson attitudes towards free-farrowing systems. The primary aim of the experiment was to examine stockperson attitudes towards the two lactation housing systems, and so an opinion-based assessment of sow welfare was developed to be included in the survey whilst not distracting from other included questions. The score did consider qualitative behavioural assessment (QBA) and used descriptors of emotions and groupings reported previously (38). Whilst the current investigation did apply similar descriptors of emotion, QBA was too complex to be included in the survey. This methodology still remains to be applied to periparturient sows (39) and so should be explored in further work.

While it is well known that education can help to improve stockperson attitude towards the animals they tend (24), the current study is the first to examine how stockperson attitudes to different housing systems were related to their perceptions of sow welfare in these housing systems. Stockpeople that scored a sow higher for welfare in a MR believed they were confident enough in their understanding of pig behaviour to work safely in these systems. This finding suggests that transition to any alternative husbandry system could be increased if we improve stockperson understanding of animal behaviour, and in so doing shifting attitudes towards safety. Stockpeople with positive attitudes towards the use of farrowing crates (FC defenders) were more likely to rate the welfare of a sow in a farrowing crate as high, to avoid interactions with difficult pigs and were less confident in their own knowledge of sow behaviour. Control beliefs refer to how a person’s perception of their ability affects their actions (15), with a person’s doubt in their ability to work safely alongside free-farrowing sows being a limiting factor to the acceptance of the MR. A simple way to overcome this fear could be addressed by the sharing of knowledge through targeted training programs. There are numerous examples describing the positive impact training can play on an attitudinal shift (9, 16–20). Training has been proven to improve positive stockperson behaviour, leading to an increase in both welfare and production outcomes for the animal (11, 21–26).

There has never been a widely adopted training program dedicated to the farrowing sow, nor the stockpeople that work in these systems. Stockpeople on pig farms do work with unconfined sows, commonly during mating and gestation when exhibited behaviours are vastly different to those observed during the periparturient period. The use of confinement in the form of a FC has prevented the adequate expression of some of these behaviours, in addition to reducing the level of animal contact the stockperson encounters. Free-farrowing facilitates behaviours and human-animal interaction that even highly experienced stockpeople working in crated systems will have unlikely encountered. The risk to stockpersons working with unconfined sows at farrowing and during lactation stem from maternal aggression, whereby the sow is reactive towards humans, standing quickly, vocalizing and may launch and bite (40). More generalised training programs that focus on ‘pig behaviour’ will need refocusing towards farrowing and lactating sow behaviour to reduce fear and increase confidence in stockpeople working with unconfined sows.

The second key area that requires attention to shift the attitudes of stockpeople towards supporting free-farrowing is safety. When stockpeople were comfortable in their knowledge of sow behaviour, they felt safe working in the MR system. Stockperson safety is or should be the first consideration in the adoption process of free-farrowing systems, however there is little scientific literature addressing safety in free-farrowing systems. Baxter et al. (2) discusses key design elements that can help to improve stockperson safety, however most of the scientific literature relates to temporary confinement systems and not free-farrowing accommodation. Despite the lack of literature some European countries (Sweden and Germany) have put in place additional regulations regarding stockperson safety specifically around farrowing sows, whilst this is a step in the right direction these decisions have been largely based on case studies and interviews (2), not scientific outcomes. Training programs that focus on stockperson safety during pig transport have been developed after the identification that animal contact is the leading cause of injury on farms (41). A priority should also be a better understanding of the key stockperson behaviours and human-animal interactions which affect sow welfare in free-farrowing systems. Once these key behaviours are identified, the relevant stockperson attitudes can be targeted to increase or reduce these behaviours. As seen in the use of ProHand programs, this is also likely to improve stockperson job satisfaction and work motivation (21). A better understanding of what stockpeople feel contributes to a safe working environment in free-farrowing (outside understanding sow behaviour discussed above) requires further evaluation. Identifying the risks to stockpeople that could result in injury in free-farrowing systems should be systematically assessed and minimised. The implementation of a structured safety assessment procedure in free-farrowing would not only ensure stockpeople feel and are indeed safe but could also have residual benefits for animal welfare, performance and job satisfaction.

Stockpeople that were more satisfied in their job were more likely to rate sow welfare as higher in a MR. These were stockpeople who were more likely to undertake training in their own time if provided and found their job interesting. It is well understood that job satisfaction of stockpeople results in higher standards of animal welfare (25, 32, 42), and that the provision of training increases their likelihood to remain in the job (17). Therefore, stockpeople who are more engaged in their work and are eager to learn appear to be more open-minded about future industry changes, such as free-farrowing, and are more likely to be retained in the industry. Given that those termed FC defenders deemed crates as a necessity, specialized training programs are an essential and proven way of modifying attitudes and may help to improve attitudes towards free-farrowing systems. Farrowing crate defenders had less confidence in their ability to work safely in this environment, highlighting a lack of knowledge or experience working with free-farrowing sows. Stockpeople who scored sow welfare as high in a FC believed that piglet welfare was more important than sow welfare, this is likely due to the education that has occurred around the introduction of the FC and its ability to improve piglet safety. Whilst the farrowing crate has provided benefits for piglet survival, it does impose physical restrictions impacting sow welfare that result in socially unsustainable practices (3, 5, 27). The development of future training programs should therefore have a dual focus of not just *how* to work safely in a free-farrowing environment, but also to educate stockpeople on the reasons *why* as an industry there is a need to move towards systems that provide improved welfare and allow the animals we care for to create ‘a life worth living’.

The results of this study suggest that there is a fear of working in free-farrowing systems that stem from a lack of experience and knowledge of maternal sow behaviour. Stockpeople that viewed sow welfare in a MR more positively were confident in their knowledge of sow behaviour and felt this contributed to a safer working environment, whilst those that supported sow welfare in a FC believed the crate was, and would remain, an essential part of pig farming. The development of a free-farrowing training program, focused on educating stockpeople on not just *how* to work in these environments, but *why* they are beneficial for the sow has the potential to aid in their acceptance and the ability to improve job satisfaction and stockperson retention. This will enable greater engagement in the issue of sow and piglet welfare around farrowing and lactation and prepare stockpeople with increased confidence in their own abilities.

## Data Availability

The raw data supporting the conclusions of this article will be made available by the authors, without undue reservation.

## References

[1] Robertson JB Laird R Hall JKS Forsyth RJ Thomson JM Walker-Love J. A comparison of two indoor farrowing systems for sows. Anim Sci. (1966) 8:171–8. doi: 10.1017/S0003356100034553

[2] Baxter EM Moustsen VA Goumon S Illmann G Edwards SA. Transitioning from crates to free farrowing: a roadmap to navigate key decisions. Front Vet Sci. (2022) 9:998192. doi: 10.3389/fvets.2022.998192, PMID: 36452143 PMC9701704

[3] Glencorse D Plush KJ Hazel S D’Souza DN Hebart M. Impact of non-confinement accommodation on farrowing performance: a systematic review and meta-analysis of farrowing crates versus pens. Animals. (2019) 9:957. doi: 10.3390/ani9110957, PMID: 31726676 PMC6912515

[4] Goumon S Illmann G Moustsen VA Baxter EM Edwards SA. Review of temporary crating of farrowing and lactating sows. Front Vet Sci. (2022) 9:811810. doi: 10.3389/fvets.2022.811810, PMID: 35372543 PMC8969568

[5] Vandresen B Hötzel MJ. Pets as family and pigs in crates: public attitudes towards farrowing crates. Appl Anim Behav Sci. (2021) 236:105254. doi: 10.1016/j.applanim.2021.105254

[6] Bergstra TJ Gremmen B Stassen EN. Moral values and attitudes toward Dutch sow husbandry. J Agric Environ Ethics. (2015) 28:375–401. doi: 10.1007/s10806-015-9539-x

[7] Boogaard BK Boekhorst LJS Oosting SJ Sørensen JT. Socio-cultural sustainability of pig production: citizen perceptions in the Netherlands and Denmark. Livest Sci. (2011) 140:189–200. doi: 10.1016/j.livsci.2011.03.028

[8] Ryan EB Fraser D Weary DM. Public attitudes to housing systems for pregnant pigs. PLoS One. (2015) 10:e0141878. doi: 10.1371/journal.pone.0141878, PMID: 26559417 PMC4641725

[9] Coleman GJ McGregor M Hemsworth PH Boyce J Dowling S. The relationship between beliefs, attitudes and observed behaviours of abattoir personnel in the pig industry. Appl Anim Behav Sci. (2003) 82:189–200. doi: 10.1016/S0168-1591(03)00057-1

[10] Gocsik É Lansink AGJMO Saatkamp HW. Mid-term financial impact of animal welfare improvements in Dutch broiler production. Poult Sci. (2013) 92:3314–29. doi: 10.3382/ps.2013-03221, PMID: 24235244

[11] Sinclair M Zito S Phillips CJC. The impact of stakeholders’ roles within the livestock industry on their attitudes to livestock welfare in southeast and East Asia. Animals. (2017) 7:6. doi: 10.3390/ani7020006, PMID: 28125058 PMC5332927

[12] Waiblinger S Boivin X Pedersen V Tosi MV Janczak AM Visser EK. Assessing the human–animal relationship in farmed species: a critical review. Appl Anim Behav Sci. (2006) 101:185–242. doi: 10.1016/j.applanim.2006.02.001

[13] Borgen SO Skarstad GA. Norwegian pig farmers' motivations for improving animal welfare. Br Food J. (2007) 109:891–905. doi: 10.1108/00070700710835705

[14] Breuer K Hemsworth PH Barnett JL Matthews LR Coleman GJ. Behavioural response to humans and the productivity of commercial dairy cows. Appl Anim Behav Sci. (2000) 66:273–88. doi: 10.1016/S0168-1591(99)00097-0, PMID: 10700627

[15] Ajzen I. The theory of planned behavior. Organ Behav Hum Decis Process. (1991) 50:179–211. doi: 10.1016/0749-5978(91)90020-T

[16] Ceballos MC Sant'Anna AC Boivin X de Oliveira Costa F Carvalhal MVL da Costa MP. Impact of good practices of handling training on beef cattle welfare and stockpeople attitudes and behaviors. Livest Sci. (2018) 216:24–31. doi: 10.1016/j.livsci.2018.06.019

[17] Coleman GJ Hemsworth PH Hay M Cox M. Modifying stockperson attitudes and behaviour towards pigs at a large commercial farm. Appl Anim Behav Sci. (2000) 66:11–20. doi: 10.1016/S0168-1591(99)00073-8

[18] Crawford SM. Improving the attitudes and behavior of stockpersons toward pigs and the subsequent influence on animal behavior and production characteristics of commercial finishing pigs in Ohio. Columbus, OH, United States: The Ohio State University (2011).

[19] Descovich K Li X Sinclair M Wang Y Phillips CJC. The effect of animal welfare training on the knowledge and attitudes of abattoir stakeholders in China. Animals. (2019) 9:989. doi: 10.3390/ani9110989, PMID: 31752147 PMC6912204

[20] Leon AF Sanchez JA Romero MH. Association between attitude and empathy with the quality of human-livestock interactions. Animals. (2020) 10:1304. doi: 10.3390/ani10081304, PMID: 32751442 PMC7459475

[21] Coleman GJ Hemsworth PH. Training to improve stockperson beliefs and behaviour towards livestock enhances welfare and productivity. Rev Sci Tech. (2014) 33:131–7. doi: 10.20506/rst.33.1.2257, PMID: 25000785

[22] Hemsworth PH Barnett JL Coleman GJ Hansen C. A study of the relationships between the attitudinal and behavioural profiles of stockpersons and the level of fear of humans and reproductive performance of commercial pigs. Appl Anim Behav Sci. (1989) 23:301–14. doi: 10.1016/0168-1591(89)90099-3

[23] Hemsworth PH Coleman GJ. A model of stockperson-animal interactions and their implications for animals In: Hemsworth PH Coleman GJ, editors. Human-livestock interactions. (CABI, Wallingford, UK: The Stockperson and the Productivity and Welfare of Intensively Farmed Animals). (2011) 91–106.

[24] Hemsworth PH Coleman GJ Barnett JL. Improving the attitude and behaviour of stockpersons towards pigs and the consequences on the behaviour and reproductive performance of commercial pigs. Appl Anim Behav Sci. (1994) 39:349–62. doi: 10.1016/0168-1591(94)90168-6

[25] Hemsworth PH Coleman GJ Barnett JL Borg S Dowling S. The effects of cognitive behavioral intervention on the attitude and behavior of stockpersons and the behavior and productivity of commercial dairy cows. J Anim Sci. (2002) 80:68–78. doi: 10.2527/2002.80168x, PMID: 11831530

[26] Munoz CA Coleman GJ Hemsworth PH Campbell AJD Doyle RE. Positive attitudes, positive outcomes: the relationship between farmer attitudes, management behaviour and sheep welfare. PLoS One. (2019) 14:e0220455. doi: 10.1371/journal.pone.0220455, PMID: 31365546 PMC6668801

[27] Plush K Lines D Staveley L D’Souza D van Barneveld R. A five domains assessment of sow welfare in a novel free farrowing system. Font Anim Sci. (2024) 11:1339947. doi: 10.3389/fvets.2024.1339947, PMID: 39229595 PMC11370643

[28] Coleman G Jongman E Greenfield L Hemsworth P. Farmer and public attitudes toward lamb finishing systems. J Appl Anim Welf Sci. (2016) 19:198–209. doi: 10.1080/10888705.2015.1127766, PMID: 26882113

[29] Coleman G Rohlf V Toukhsati S Blache D. Public attitudes predict community behaviours relevant to the pork industry. Anim Prod Sci. (2017) 58:416–23. doi: 10.1071/AN16776, PMID: 40460249

[30] Coleman G Toukhsati S. Consumer attitudes and behaviour relevant to the red meat industry. Meat and Livestock Australia Limited: North Sydney, NSW (2006).

[31] Hemsworth L Rice M Hemsworth P Coleman G. Telephone survey versus panel survey samples assessing knowledge, attitudes and behavior regarding animal welfare in the red meat industry in Australia. Front Psychol. (2021) 12:581928. doi: 10.3389/fpsyg.2021.581928, PMID: 33897517 PMC8060561

[32] Hemsworth PH Coleman GJ. Human-animal interactions and animal productivity and welfare In: Human-livestock interactions: The stockperson and the productivity and welfare of intensively farmed animals. 2nd ed (2011). 47–83.

[33] Pallant J. SPSS survival manual McGraw-hill education (UK). Maidenhead: Open University Press (2013).

[34] DeVellis RF. Scale development: theory and applications. J Int Acad Res. (2003) 10:23–41. doi: 10.1177/109821409301400212

[35] Tabachnick BG Fidell LS. Using multivariate statistics. 5th ed. Boston, MA: Allyn & Bacon/Pearson Education (2007).

[36] Guy JH Cain PJ Seddon YM Baxter EM Edwards SA. Economic evaluation of high welfare indoor farrowing systems for pigs. Anim Welfare UFAWJ. (2012) 21:19–24. doi: 10.7120/096272812X13345905673520

[37] Nowland TL van Wettere WHEJ Plush KJ. Allowing sows to farrow unconfined has positive implications for sow and piglet welfare. Appl Anim Behav Sci. (2019) 221:104872. doi: 10.1016/j.applanim.2019.104872

[38] Wemelsfelder F Hunter EA Mendl MT Lawrence AB. The spontaneous qualitative assessment of behavioural expressions in pigs: first explorations of a novel methodology for integrative animal welfare measurement. Appl Anim Behav Sci. (2000) 67:193–215. doi: 10.1016/S0168-1591(99)00093-3, PMID: 10736529

[39] Vandresen B Chou JY Hötzel MJ. How is pig welfare assessed in studies on farrowing housing systems? A systematic review. Appl Anim Behav Sci. (2024) 275:106298. doi: 10.1016/j.applanim.2024.106298, PMID: 40521292

[40] Marchant JN. Piglet- and stockperson-directed sow aggression after farrowing and the relationship with a pre-farrowing, human approach test. Appl Anim Behav Sci. (2002) 75:115–32. doi: 10.1016/S0168-1591(01)00170-8

[41] Langley RL Morrow WEM. Livestock handling—minimizing worker injuries. J Agromedicine. (2010) 15:226–35. doi: 10.1080/1059924X.2010.486327, PMID: 20665308

[42] Dawkins MS. Animal welfare and efficient farming: is conflict inevitable? Anim Prod Sci. (2016) 57:201–8. doi: 10.1071/AN15383, PMID: 40460249

