# Supplementary material for: Stockperson attitudes towards Maternity Rings and farrowing crates
Source: Front Vet Sci. 2025 Jul 23;12:1579263. doi: 10.3389/fvets.2025.1579263 (PMC12325031; doi:10.3389/fvets.2025.1579263)
Supplement: Supplementary file 1 [file Table_1.docx]

| **Question** | **Scale Type** | **n** | **Mean** | **SD** |
| --- | --- | --- | --- | --- |
| Pigs are gluttons | Agreement | 83 | 2.94 | 1.282 |
| Pigs are dirty | Agreement | 85 | 2.87 | 1.213 |
| Pigs are easy to work with | Agreement | 85 | 3.56 | 0.993 |
| Pigs are friendly to people | Agreement | 83 | 3.84 | 0.994 |
| Pigs are easily frightened | Agreement | 83 | 3.60 | 1.178 |
| Pigs are intelligent | Agreement | 85 | 4.04 | 1.085 |
| Pigs are mischievous | Agreement | 84 | 3.52 | 1.024 |
| Pigs are curious | Agreement | 83 | 4.29 | 0.904 |
| I frequently pat pigs that are not ‘on heat’ | Agreement | 83 | 3.76 | 1.111 |
| I frequently pat ‘on heat’ pigs | Agreement | 84 | 3.39 | 1.162 |
| I find ‘on heat’ pigs frustrating | Agreement | 84 | 2.82 | 1.184 |
| I frequently talk to pigs that are not ‘on heat’ | Agreement | 85 | 3.32 | 1.187 |
| I frequently talk to ‘on heat’ pigs | Agreement | 85 | 3.31 | 1.185 |
| I find talking ‘on heat’ pigs does not help with daily tasks | Agreement | 82 | 3.04 | 1.181 |
| I frequently pat calm/happy sows in the farrowing house | Agreement | 84 | 3.90 | 1.060 |
| I frequently pat protective sows in the farrowing house | Agreement | 83 | 2.89 | 1.230 |
| I avoid protective sows in the farrowing house | Agreement | 84 | 2.96 | 1.156 |
| I frequently talk to calm/happy sows in the farrowing house | Agreement | 85 | 3.76 | 1.130 |
| I frequently talk to protective sows in the farrowing house | Agreement | 85 | 3.55 | 1.160 |
| I find talk to protective sows in the farrowing house does not help with daily tasks | Agreement | 85 | 2.86 | 1.167 |
| Rate the physical effort to move pigs on heat | Difficulty | 84 | 2.75 | 0.992 |
| Rate the verbal effort to move pigs on heat | Difficulty | 82 | 2.88 | 0.961 |
| Rate the perceived difficulty to move pigs on heat | Difficulty | 84 | 2.76 | 1.001 |
| Rate the physical effort to perform routine husbandry in farrowing around protective sows | Difficulty | 83 | 2.89 | 0.937 |
| Rate the verbal effort to perform routine husbandry in farrowing around protective sows | Difficulty | 85 | 2.98 | 0.926 |
| Rate the perceived difficulty to perform routine husbandry in farrowing around protective sows | Difficulty | 85 | 2.85 | 0.880 |
| Rate the physical effort required to move non estrous pigs | Difficulty | 85 | 3.59 | 0.955 |
| Rate the verbal effort required to move non estrous pigs | Difficulty | 84 | 3.56 | 0.910 |
| Rate the perceived difficulty to move non estrous pigs | Difficulty | 84 | 3.40 | 0.933 |
| Rate the physical effort to perform routine husbandry in farrowing around calm/friendly sows | Difficulty | 85 | 4.02 | 0.988 |
| Rate the verbal effort to perform routine husbandry in farrowing around calm/friendly sows | Difficulty | 85 | 4.01 | 0.994 |
| Rate the perceived difficulty to perform routine husbandry in farrowing around calm/friendly sows | Difficulty | 85 | 3.92 | 0.903 |
| Is your job boring? | Agreement | 86 | 2.14 | 1.108 |
| Do you think you’ll be in the pig industry in 5 years? | Likelihood | 86 | 3.74 | 1.457 |
| Do you think you’ll be in the pig industry in 10 years? | Likelihood | 86 | 3.19 | 1.435 |
| When you go away for holidays how likely are you to go with a family member? | Likelihood | 84 | 4.23 | 1.176 |
| How likely is it that family/close friend to supervise your children/pet when you’re not able to? | Likelihood | 84 | 3.76 | 1.276 |
| How likely are you to pay for services to supervise your children/pet when you’re not able to? | Likelihood | 84 | 3.12 | 1.339 |
| I look forward to smoko/lunch breaks? | Agreement | 86 | 3.98 | 1.106 |
| I look forward to the end of the working day? | Agreement | 86 | 4.00 | 1.085 |
| I often have to work in cramped conditions | Agreement | 82 | 2.83 | 1.063 |
| The air is clean at work | Agreement | 86 | 2.52 | 1.145 |
| How often do you discuss work methods during smoko/lunch breaks? | Likelihood | 86 | 3.28 | 1.081 |
| Would you attend training courses in your own time if they were available? | Likelihood | 86 | 3.33 | 1.491 |
| I know a lot about diseases in pigs | Agreement | 86 | 3.34 | 1.025 |
| I know a lot about factors which affect reproduction in pigs | Agreement | 86 | 3.63 | 1.063 |
| I understand pig behaviour well | Agreement | 86 | 4.03 | 0.676 |
| How would you classify the sows demeanour? | Descriptor | 84 | 3.13 | 1.210 |
| How well do you think the sow is able to express her natural behaviour? | Descriptor | 82 | 3.10 | 1.182 |
| How would you classify the sows demeanour? | Descriptor | 80 | 3.73 | 1.055 |
| How well do you think the sow is able to express her natural behaviour? | Descriptor | 81 | 3.74 | 1.022 |
| How would you rate the welfare of this sow? | Descriptor | 82 | 3.00 | 1.111 |
| How easily is this sow able to perform the desired behaviour? | Descriptor | 79 | 3.06 | 1.078 |
| How do you think this sow feels? | Descriptor | 79 | 3.13 | 1.125 |
| How would you rate the welfare of this sow? | Descriptor | 79 | 3.68 | 1.007 |
| How easily is this sow able to perform the desired behaviour? | Descriptor | 79 | 3.72 | 0.999 |
| How do you think this sow feels? | Descriptor | 80 | 3.84 | 1.024 |
| How would you rate the welfare of this sow? | Descriptor | 77 | 2.70 | 1.065 |
| How easily is this sow able to perform the desired behaviour? | Descriptor | 77 | 2.48 | 1.096 |
| How do you think this sow feels? | Descriptor | 76 | 2.74 | 1.124 |
| How would you rate the welfare of this sow? | Descriptor | 79 | 3.92 | 1.047 |
| How easily is this sow able to perform the desired behaviour? | Descriptor | 79 | 4.05 | 0.973 |
| How do you think this sow feels? | Descriptor | 77 | 4.00 | 0.960 |
| How do you think this interaction makes the sow feel? | Descriptor | 78 | 2.60 | 1.085 |
| How well is the sow able to interact with her piglets? | Descriptor | 78 | 2.64 | 1.032 |
| How do you think this interaction makes the sow feel? | Descriptor | 76 | 3.84 | 1.033 |
| How well is the sow able to interact with her piglets? | Descriptor | 77 | 3.90 | 1.107 |
| I prefer working with sows in farrowing crates | Agreement | 85 | 3.74 | 0.966 |
| I prefer working with sows in farrowing pens | Agreement | 84 | 3.06 | 1.134 |
| I feel safe working in farrowing crates | Agreement | 85 | 3.86 | 1.014 |
| I feel safe working in farrowing pens | Agreement | 84 | 2.96 | 1.092 |
| I prefer weaning in farrowing crates | Agreement | 84 | 3.58 | 1.214 |
| I prefer weaning in farrowing pens | Agreement | 84 | 3.02 | 1.212 |
| How difficult is assisting a farrowing sow in a crate? | Difficulty | 82 | 3.54 | 1.178 |
| How difficult is assisting a farrowing sow in a pen? | Difficulty | 83 | 2.78 | 1.169 |
| How difficult is weaning in a crate? | Difficulty | 83 | 3.31 | 1.115 |
| How difficult is weaning in a pen? | Difficulty | 82 | 2.96 | 1.242 |
| I believe farrowing crates are better for the sow’s welfare | Importance | 83 | 2.71 | 1.077 |
| I believe farrowing pens are better for the sow’s welfare | Importance | 84 | 3.87 | 1.015 |
| I believe farrowing crates are better for the piglet’s welfare | Importance | 84 | 3.39 | 1.193 |
| I believe farrowing pen are better for the piglet’s welfare | Importance | 84 | 3.31 | 1.130 |
| I believe the sow welfare is more important than piglet welfare | Importance | 83 | 2.88 | 1.005 |
| I believe piglet welfare is more important than sow welfare | Importance | 85 | 2.95 | 1.057 |
| I believe I understand sow behaviour well enough to work safely in farrowing pens | Agreement | 84 | 3.93 | 0.875 |
| I believe detecting sows that need medical intervention is easier in a farrowing crate | Agreement | 85 | 3.47 | 1.119 |
| I believe detecting sows that need medical intervention is easier in a farrowing crate | Agreement | 84 | 3.31 | 1.053 |
| I would be proud to show my friends/family around a farrowing house with only farrowing crates | Agreement | 83 | 3.05 | 1.092 |
| I would be proud to show my friends/family around a farrowing house with only farrowing crates | Agreement | 84 | 3.43 | 0.973 |
| I believe farrowing crates will always be necessary | Agreement | 85 | 3.16 | 1.122 |
| I believe farrowing pens are the future of the industry | Agreement | 84 | 3.55 | 1.069 |
